# Isolation and Genome-Based Characterization of *Bacillus velezensis* AN6 for Its Biocontrol Potential Against Multiple Plant Pathogens

**DOI:** 10.3390/microorganisms13122701

**Published:** 2025-11-27

**Authors:** Liping Yang, Anyu Gu, Wei Deng, Shu Che, Jianhua Zhang, Jinwen Zhang, Limei Kui, Jian Tu, Wei Dong, Hua An, Junjiao Guan, Jiaqin Fan, Xiqiong Shen, Xiaolin Li

**Affiliations:** 1 Food Crops Research Institute, Yunnan Academy of Agricultural Sciences (YAAS), Kunming 650205, China; yangliping@yaas.org.cn (L.Y.); ynzycxtd_gu@sina.cn (A.G.); dengw@yaas.org.cn (W.D.); zhjhua6748@163.com (J.Z.); zjwen@yaas.org.cn (J.Z.); klm@yaas.org.cn (L.K.); tj@yaas.org.cn (J.T.); dwei@yaas.org.cn (W.D.); anh@yaas.org.cn (H.A.); guanjunjiao@163.com (J.G.); 15877998572@163.com (X.S.); 2 Laboratory of Bacteriology, Department of Plant Pathology, Nanjing Agricultural University, Nanjing 210095, China; shuche0523@163.com (S.C.); fanjq@njau.edu.cn (J.F.); 3 Plant Protection Institute, Jiangxi Academy of Agricultural Sciences, Nanchang 330200, China

**Keywords:** *Bacillus velezensis*, *Xanthomonas oryzae* pv. *oryzae* (*Xoo*), plant pathogens, biological control, broad-spectrum antagonistic activities, whole-genome sequencing

## Abstract

Biological control is an effective and environmentally friendly strategy for managing plant diseases. In this study, a broad-spectrum antagonistic bacterium, designated strain AN6, was isolated from rice plants and exhibited potent inhibitory activity against a variety of phytopathogens. In Oxford cup assays, AN6 suppressed the growth of *Xanthomonas oryzae* pv. *oryzae* (*Xoo*) by 73.60%, and its cell-free culture filtrate caused pronounced morphological deformation in the bacterial cells. Further *in vitro* assays, including dual-culture assays, volatile organic compound (VOC) assays, and cell-free supernatant (CFS) assays, demonstrated that AN6 also exerted strong antifungal effects against several pathogenic fungi. In addition, the strain was found to produce proteases and siderophores, which may contribute to its antagonistic capabilities. Taxonomic identification based on morphological traits, 16S rRNA and *gyrA* gene sequencing, average nucleotide identity (ANI), in silico DNA–DNA hybridization (*is*DDH), and phylogenetic analysis classified strain AN6 as *Bacillus velezensis*. Whole-genome sequencing revealed that AN6 harbors a 3,929,788 bp genome comprising 4025 protein-coding genes with a GC content of 46.50%. Thirteen biosynthetic gene clusters (BGCs) associated with the production of secondary metabolites—such as nonribosomal peptides, polyketides, and dipeptide antibiotics—were identified. The pot experiment further validated the biocontrol potential of AN6, achieving an 80.49% reduction in rice bacterial blight caused by *Xanthomonas oryzae* pv. *oryzae*. Collectively, these results indicate that *B. velezensis* AN6 is a promising candidate for development as a highly effective biocontrol agent for the integrated management of diverse plant diseases.

## 1. Introduction

Rice (*Oryza sativa* L.) is not only a staple food for more than half of the global population, but also a key crop in many countries, particularly in Asia, where it is essential for food security and economic stability [1,2]. However, rice production faces numerous challenges, from both biotic and abiotic stresses, which significantly affect yield and quality. Among these, bacterial leaf blight (BLB), caused by the Gram-negative bacterium *Xanthomonas oryzae* pv. *oryzae* (*Xoo*), stands out as one of the most destructive bacterial diseases in rice cultivation; it is also listed as one of the top ten plant pathogenic bacteria [3,4]. In addition, rice bacterial blight is also a major rice disease in Asian countries. High-yield rice varieties are highly susceptible to the disease. In severely endemic areas, rice bacterial blight can cause yield losses of up to 60%, primarily by affecting the photosynthetic capacity of the plant. Symptoms typically include the appearance of water-soaked lesions that eventually turn into necrotic streaks, leading to premature leaf senescence and reduced grain filling [5]. Conventional management strategies for BLB include the use of resistant rice cultivars, chemical pesticides, and agronomic practices such as crop rotation [6,7,8]. However, these approaches often come with significant limitations. For example, the widespread use of chemical pesticides has raised concerns about environmental pollution, human health risks, and the development of pesticide-resistant bacterial strains [9]. In addition, the variability in *Xoo* populations and the emergence of new pathogenic strains complicate the effectiveness of resistant cultivars. Consequently, these traditional control measures are often insufficient for long-term disease management. Sustainable and eco-friendly approaches, such as biological control, are garnering increasing amounts of attention.

Biological control can inhibit the growth of harmful organisms through the use of antagonistic microorganisms such as bacteria, fungi, and actinomycetes, providing an environmentally friendly control method for agricultural production. Bio-control agents made from biocontrol bacteria are being increasingly applied in sustainable agriculture. The most studied biocontrol bacteria are *Bacillus* species. Inoculants derived from *Bacillus* bacteria have been proven to be environmentally friendly alternatives to chemical pesticides [10]. The *Bacillus* species that exhibit antagonistic activity against pathogenic fungi include *B. subtilis*, *B. amyloliquefaciens*, *B. cereus*, *B. mycoides*, *B. pumilus*, *B. pasteurii*, and *B. sphaericus*, among others [11]. It has been reported that these bacteria defend against pathogens by producing antibiotic metabolites, antimicrobial compounds, and by inducing or stimulating systemic resistance in plants [12,13,14,15,16]. Among these, *B. amyloliquefaciens* can produce lipopeptides (LPs) and has shown significant inhibitory activity against pathogens such as *Alternaria panax*, *Botrytis cinerea*, *Colletotrichum orbiculare*, *Penicillium digitatum*, *Pyricularia grisea*, *Sclerotinia sclerotiorum*, and *Acidovorax oryzae* [17,18]. Furthermore, *B. amyloliquefaciens* SQR9 inhibits the growth of *Fusarium oxysporum* by producing antibiotics, protecting cucumber plants from invasion [19]. *B. subtilis* ME488 produces antibiotics that strongly inhibit *F. oxysporum* f. sp. *cucumerinum*, the pathogen that causes wilt in cucumbers [20]. *B. subtilis* B006 produces surfactants that inhibit the pathogen in soil by 42.9% [21]. The lipopeptide extract from *B. amyloliquefaciens* SS-12.6 has shown significant potential for biological control against *Pseudomonas syringae* pv. *aptata*, the pathogen that causes leaf spot in beets [22]. Additionally, *B. pumilus* T4 induces plant resistance to *P. syringae* through a novel or mutated pathway [23]. Moreover, systemic resistance induced by *Bacillus* biocontrol bacteria can protect plants from damage caused by leaf spot diseases, viral infections, crown rot, root knot nematodes, and stem rot diseases [11].

*B. velezensis* is a potential biocontrol agent that was first isolated by Ruiz-García et al. from brackish water samples collected from the Vélez River [24]. *B. velezensis* can produce a variety of antimicrobial metabolites, including lipopeptide antibiotics (surfactin, fengycin, and bacillomycin D), polyketides (macrolactin, bacillaene, difficidin, and oxydifficidin), and peptides (plantazolicin, amylocyclicin, and bacilysin) [25]. Studies have shown that *B. velezensis* can significantly inhibit several pathogenic microorganisms, such as *Botrytis cinerea* [26], *Colletotrichum gloeosporioides* [27,28,29,30], *Phytophthora infestans* [31], *Agrobacterium tumefaciens* [32,33], *Ralstonia solanacearum* [34], *F. oxysporum* [16,34,35], *Xanthomonas euvesicatoria* [36] (which causes bacterial spot in peppers), *Xanthomonas oryzae* pv. *oryzae* [37] (which causes bacterial blight in rice), and *Phytophthora nicotianae* [38] (which causes black shank in tobacco). These pathogens are responsible for diseases affecting various crops, ornamental plants, and fruit trees. Additionally, *B. velezensis* can induce plant tolerance to abiotic stress and promote plant growth [39]. Whether used as a biocontrol agent or a plant growth regulator, *B. velezensis* holds great potential for widespread application in agriculture.

This study aimed to investigate the biocontrol potential of *B. velezensis* AN6 and to determine its genome sequence. Bioassays indicated that AN6 could serve as a biocontrol agent against rice bacterial blight (*Xanthomonas oryzae* pv. *oryzae*) and that it also has broad-spectrum antifungal activity. Phenotypic assays and genomic analysis combined with secondary metabolite prediction further indicated that the disease-suppressive capacity of AN6 may be associated with its ability to produce proteases, siderophores, volatile organic compounds (VOCs), and antimicrobial secondary metabolites. Overall, these results indicate that *B. velezensis* AN6 represents a valuable biocontrol resource with promising potential for environmentally friendly management of plant diseases.

## 2. Materials and Methods

### 2.1. Bacterium and Pathogens Used in This Study

The strain AN6 was isolated from the rice leaves collected at a base (100.13° E, 25.85° N) in Dali City, Yunnan Province, China. It is currently preserved at the China Center for Type Culture Collection (CCTCC) under accession number CCTCC M 20231886. The *Xanthomonas oryzae* pv. *oryzae* strains (X1, X2, X3, X12, LC2, and LC10) and other phytopathogenic fungi used in this study are summarized in Table S1.

### 2.2. Isolation and Morphological Observation of Endophytic Bacteria

The isolation and culture of endophytic bacteria from rice leaves were performed according to previously published methods with slight modifications [40]. The pure culture strain was then numbered sequentially. Single colonies of the pure culture strain were inoculated into NB liquid medium and incubated at 28 °C with shaking at 180 rpm for 12 h. After incubation, 1 mL of the bacterial suspension was mixed with 1 mL of 50% sterile glycerol and stored at −80 °C for long-term preservation.

### 2.3. Screening of Biocontrol Strains with Resistance to Xanthomonas oryzae pv. oryzae

The Oxford cup method, previously described in [41], was used to evaluate the inhibitory activity of the endophytic bacterial isolates (hereafter referred to as “test strains”) against *Xoo*. Briefly, the test strains and *Xoo* isolates (X1, X2, X3, X12, LC2, and LC10) were cultured in NB at 28 °C and 180 rpm until OD_600_ ≈ 1.0. Different *Xoo* cells were mixed with NA medium (1:100, *v*/*v*) to prepare indicator plates. A sterile 6 mm Oxford cup was placed in the center of each plate and 50 µL of the test strain suspension was added. Plates were incubated at 28 °C for 48 h. The diameter of the inhibition zone was measured using the cross method, and the inhibition rate was calculated using the following formula: Inhibition rate (%) = [(Inhibition zone diameter − 6)/Inhibition zone diameter] × 100%. We screened for the strain with the strongest antibacterial activity against *Xoo*.

### 2.4. Morphological and Biological Characteristics of the Strain AN6

Morphological and biological analyses of strain AN6 were conducted. The morphological characteristics of the AN6 strain were observed using a Biological Microscope (Olympus BX53, Shinjuku, Tokyo, Japan) and scanning electron microscope (HITACHI Regulus 8100; Hitachi Production Co., Ltd., Tokyo, Japan). Gram staining was performed using the G1060 Gram Stain Kit (Solarbio Science & Technology Co., Ltd., Beijing, China). Bacterial growth was assessed using the method described by Fan [42]. The strains were cultured overnight in NB, and then the culture was adjusted to achieve OD_600_ = 1.0. They were subsequently diluted 1:100 in NB medium, and absorbance at 600 nm was measured every 1 h. Each experiment was repeated at least three times with 3–5 replicates.

### 2.5. The Effect of Sterile AN6 Cell-Free Supernatant on the Cell Growth of Xoo

The AN6 and *Xoo* LC2 strains were independently inoculated into NB liquid medium and incubated at 28 °C with shaking at 180 rpm until the OD_600_ reached approximately 1.0. A total of 1 mL of AN6 suspension was then centrifuged at 8000 rpm for 5 min to collect the supernatant, which was filtered twice using a 0.22 μm bacterial filter to obtain sterile AN6 cell-free supernatant. In the experimental group, sterile AN6 cell-free supernatant diluted 1:100 was added to fresh NB liquid medium, along with 100 μL of the *Xoo* LC2 suspension. In the control group, 100 μL of the *Xoo* LC2 suspension was added to fresh NB liquid medium only. Both groups were incubated at 28 °C and shaken at 180 rpm until the OD_600_ of the control group reached approximately 1.0. A total of 1 mL of the bacterial culture from each group was then collected, and the cell morphology of LC2 was observed using a scanning electron microscope (HITACHI Regulus 8100; Hitachi Production Co., Ltd., Tokyo, Japan) and Transmission Electron Microscope (HITACHI SU8600; Hitachi Production Co., Ltd., Tokyo, Japan).

### 2.6. Detection of Protease Production and Iron Chelation Ability of AN6

The protease activity of AN6 was detected using the agar dilution method. The AN6 strain was cultured in NB medium for 10–12 h, and then transferred to fresh NB medium in a 1:100 dilution and cultured until the OD_600_ reached approximately 1.0. A total of 1 mL of the culture was collected, and the cells were suspended in sterile water, adjusting the OD_600_ to 1.0, and 2 µL of the suspension was dropped onto NA plates containing 10% skim milk. After the bacterial suspension dried, the plates were inverted and incubated at 28 °C for 24–36 h. The size of the clear hydrolysis zone around the colonies was then observed.

The iron chelation ability of AN6 was detected using CAS medium. CAS Solution A consisted of 1 mmol L^−1^ CAS (chrome azurol S), 4 mmol L^−1^ CTMAB (cetyltrimethylammonium bromide), and 0.1 mmol L^−1^ FeCl_3_·6H_2_O. CAS Solution B consisted of 0.1 mol L^−1^ phosphate-buffered solution, pH 7.0. CAS Solution C consisted of 2 g of sucrose, 3 g of casein hydrolysate, 20 mL of 1 mmol L^−1^ MgSO_4_, 1 mL of 1 mmol L^−1^ CaCl_2_, and 18–20 g of agar, pH 7.0. CAS Solutions A, B, and C were autoclaved at 115 °C for 15 min. Then, 5 mL of CAS Solution A and 0.5 mL of CAS Solution B were added to 100 mL of CAS Solution C, mixed well, and poured to form CAS plates. An Oxford cup was placed in the center of each CAS plate, and 50 μL of the AN6 suspension (OD_600_ = 1.0) was added. The plates were incubated at 28 °C for 2–4 days, and the transparent zone around the cup was observed.

### 2.7. Evaluation of Biocontrol Efficacy in a Light Incubator

The pot experiment was conducted in a light incubator at 28 °C and a relative humidity of 70%. Sixty-day-old rice plants of the variety “YX65” were selected for the inoculation test. The *Xoo* and AN6 cells were separately inoculated into NB medium and incubated at 28 °C with shaking at 180 rpm until the logarithmic growth phase was reached. The cultures were then centrifuged at 8000 rpm for 5 min to collect the cells, and the *Xoo* and AN6 suspensions were diluted with sterile water to an OD_600_ of approximately 1.0. *Xoo* was inoculated using the leaf clipping method, as previously described [43]. For the antagonistic bacterium AN6, it was sprayed with the diluted AN6 suspension before or after the pathogenic bacterial cells were inoculated, as described previously. The effectiveness of the treatment was tested with four different treatment groups, as detailed in Table 1. Each treatment was set up with three biological replicates, with three rice plants per replicate, and five leaves inoculated per plant. Fourteen days post-inoculation, lesion length was measured along the leaf vein from the tip of the leaf to the end of the necrotic lesion. The relative control efficacy (RCE) was calculated using the following formula: RCE (%) = [(Average lesion length in control − Lesion length in treatment)/Average lesion length in control] × 100%.

### 2.8. Evaluation of the Antifungal Spectrum of Antagonistic Strain AN6

The antifungal spectrum of strain AN6 was evaluated against a panel of pathogenic fungi, which were selected as indicator species: *M. oryzae* xg, *P. oryzae* YX, *A. tenuissima* F6, *A. alternata* F15, *F. proliferatum* A2, *F. graminearum* A5, *N. oryzae* YZJ1, *L. theobromae* F1, *N. oryzae* 46-1, *F. fujikuroi* 37-1, *E. latusicollum* A4, *N. sphaerica* YF2, *E. sorghinum* YKG1, *B. cynodontis* YSD7, *B. oryzae* YSD4, and *F. oxysporum* YM1 (Table S1). Three complementary assays were employed to assess the antagonistic activity. 

Dual-Culture Assay: Mycelial plugs (5 mm in diameter) of the indicator fungi were placed at the center of PDA plates. Five microliters of the AN6 bacterial suspensions (OD_600_ = 1.0) was spotted on each side of the fungal plug, approximately 3 cm away from the center. 

VOC Assay: The inhibitory effect of volatile metabolites was tested using the double-plate inverted method. Two sterile 9 cm Petri dishes (lids removed) were placed face-to-face. The bottom plate contained NB agar spread evenly with 200 μL of a freshly prepared bacterial suspension (OD_600_ = 1.0), while the top plate contained PDA agar inoculated in the center with a 5 mm mycelial plug of the indicator fungus. The plates were sealed together bottom-to-bottom to allow volatile exchange without physical contact between the microorganisms. 

CFS Assay: Bacterial suspensions (OD_600_ = 1.0) of AN6 were centrifuged, and the supernatant was collected and passed through a 0.22 μm sterile filter to obtain cell-free supernatant. The cell-free supernatant of AN6 was diluted 1:100 (*v*/*v*) with PDA medium to prepare assay plates. A 5 mm mycelial plug of the indicator fungus was placed at the center of each plate. For all assays, the plates inoculated with only indicator fungal blocks were used as the control group. Each treatment was repeated three times and cultured at 28 °C for 5 days. The diameter of the indicator fungal colonies in the control group and the diameter of the indicator fungal colonies in the treatment group extending toward the antagonistic bacteria were measured, and the inhibition rate was calculated according to the following formula: Inhibition rate = (diameter of the indicator fungal colony in the control group − diameter of the indicator fungal colony in the treatment group)/diameter of the indicator fungal colony in the control group × 100%.

### 2.9. Genome Sequencing and Analysis

Genomic DNA of AN6 was extracted using the SDS method [44]. The harvested DNA was detected by agarose gel electrophoresis and quantified using a Qubit^®^ 2.0 Fluorometer (Thermo Scientific, Waltham, MA, USA). Libraries for single-molecule real-time (SMRT) sequencing was constructed with an insert size of 10 kb using the SMRT bell TM Template kit, version 1.0 (Pacific Biosciences, Menlo Park, CA, USA). Finally, the library quality was assessed on the Qubit^®^ 2.0 Fluorometer (Thermo Scientific) and the insert fragment size was determined using an Agilent 2100 instrument (Agilent Technologies, Santa Clara, CA, USA). A total amount of 1 μg DNA per sample was used as input material for the DNA sample preparation. Sequencing libraries were generated using NEBNext^®^ Ultra™ DNA Library Prep Kit for Illumina (NEB, Ipswich, MA, USA) following the manufacturer’s recommendations and index codes were added to attribute sequences to each sample. Finally, the PCR products were purified (AMPure XP system, Beckman Coulter Life Sciences, Brea, CA, USA) and the size distribution of the libraries were determined using an Agilent 2100 Bioanalyzer and quantified using real-time PCR.

The whole genome of AN6 was sequenced using the PacBio Sequel platform and Illumina NovaSeq PE150 at the Beijing Novogene Bioinformatics Technology Co., Ltd. (Beijing, China). Preliminary assembly was performed using SMRT Link v5.0.1 and the variant Caller module of the SMRT Link software to correct and count the results of the preliminary assembly. The corrected assembly result, which was used as the reference sequence, was blasted against Illumina data using bwa. Based on the overlap between the head and the tail, which confirmed whether the chromosomal sequence formed a circle or not, the initial site was corrected using blast and the DNAa database.

The genome of AN6 was annotated using Prodigal (v2.6.3) [45]. Transfer RNA (tRNA) genes were predicted using tRNAscan-SE 2.0.12 [46]. Ribosome RNA (rRNA) genes were analyzed using the rRNAmmer 1.2 [47]. SMRT Link v5.0.1 [48] was used to identify methylation sites across the genome and to predict nucleotide motifs that could potentially be recognized by the associated methyltransferases. Multiple databases were used to predict gene functions including the GO (Gene Ontology), KEGG (Kyoto Encyclopedia of Genes and Genomes), COG (Clusters of Orthologous Groups), NR (Non-Redundant Protein Database), TCDB (Transporter Classification Database) and Swiss-Prot databases. The secondary metabolism gene clusters were analyzed using antiSMASH v8.0.4 [49].

### 2.10. Comparative Genomic Analysis

The 16S rRNA gene was amplified using primers 27f (5′-GAGAGTTTGATCCTGGCTCAG-3′) and 1492r (5′-ACGGATACCTTGTTACGACT-3′), while the *gyrA* gene was amplified using primers 1066r (5′-CAAGGTAATGCTCCAGGCATTGCT-3′) and 42f (5′-CAGTCAGGAAATGCGTACGTCCTT-3′). The PCR reaction system (25 μL) included 1 μL of template DNA, 0.5 μL of each primer (forward/reverse), 12 μL of PCR Buffer Mix, and 11 μL of ddH_2_O. The PCR conditions were as follows: initial denaturation at 95 °C for 5 min; followed by 30 cycles of denaturation at 94 °C for 30 s, annealing at 60 °C for 30 s, and extension at 72 °C for 90 s; and a final extension at 72 °C for 4 min. The PCR products were subjected to 1% (*w*/*v*) agarose gel electrophoresis, purified, and sequenced by Qingke Biotechnology Co., Ltd. (Beijing, China). Homology analysis of the obtained 16S rRNA and *gyrA* sequences was performed using the BLAST tool 2.15.0 on the NCBI (National Center for Biotechnology Information) website. Sequence similarity analysis was conducted using Geneious 8.0 software. A maximum likelihood phylogenetic tree for the 16S rRNA and *gyrA* genes was constructed using MEGA 11 software based on the General Time Reversible model with gamma distribution and invariant sites (GTR + G + I) and 1000 bootstrap replicates.

Average nucleotide identity (ANI) and in silico DNA–DNA hybridization (*is*DDH) analyses were conducted for strain AN6 and 16 species within the genus *Bacillus*. ANI values were calculated using JSpeciesWS (http://jspecies.ribohost.com/jspeciesws/, accessed on 15 December 2024) [50] with BLAST+ 2.2.29 (ANIb) as the alignment method. *is*DDH values were estimated using the Genome-to-Genome Distance Calculator 3.0 (https://ggdc.dsmz.de/distcalc2.php, accessed on 15 December 2024) [51], employing the recommended BLAST+ alignment and Formula 2 (identities/HSP length) for computation. Species delineation thresholds were defined as ANI ≥ 95% and *is*DDH ≥ 70% [50,52].

Based on BLAST comparisons, the protein sequences of strain AN6 were compared with those of three other strains: *B. velezensis* FZB42, *B. amyloliquefaciens* DSM7, and *B. subtilis* 168. Protein clustering was performed using Hcluster-sg (version 0.5.0) based on protein sequence similarity, with thresholds set at 50% identity and a 0.7 amino acid length difference cutoff to identify gene family clusters. A Venn diagram of homologous gene families was generated using an online tool (http://bioinformatics.psb.ugent.be/webtools/Venn/, accessed on 1 January 2024) to visualize the distribution of core and unique gene families among the strains. Phylogenetic analysis was conducted using PhyloSuite v1.2.3 software [53] based on the aligned protein sequences, and a maximum likelihood tree was constructed. Bootstrap analysis was performed with 1000 replicates to assess the robustness of the phylogenetic tree. Genomic alignment between the AN6 genome and reference genome (*B. velezensis* FZB42, *B. amyloliquefaciens* DSM7, and *B. subtilis* 168) were performed using Geneious 8.0.2 [54] and Mauve software v2.4.0 [55] with default parameters. Genomic synteny was analyzed based on the alignment results.

### 2.11. Statistical Analysis

Statistical analysis was performed using GraphPad software (version 6.0, GraphPad Software, San Diego, CA, USA), evaluated through one-way analysis of variance (ANOVA), followed by Dunnett multiple comparison post hoc tests. Significance levels are indicated as * *p* < 0.05, ** *p* < 0.01, *** *p* < 0.001, and ns (not statistically significant).

## 3. Results

### 3.1. Isolation and Screening of Bacillus

**Figure 1:**
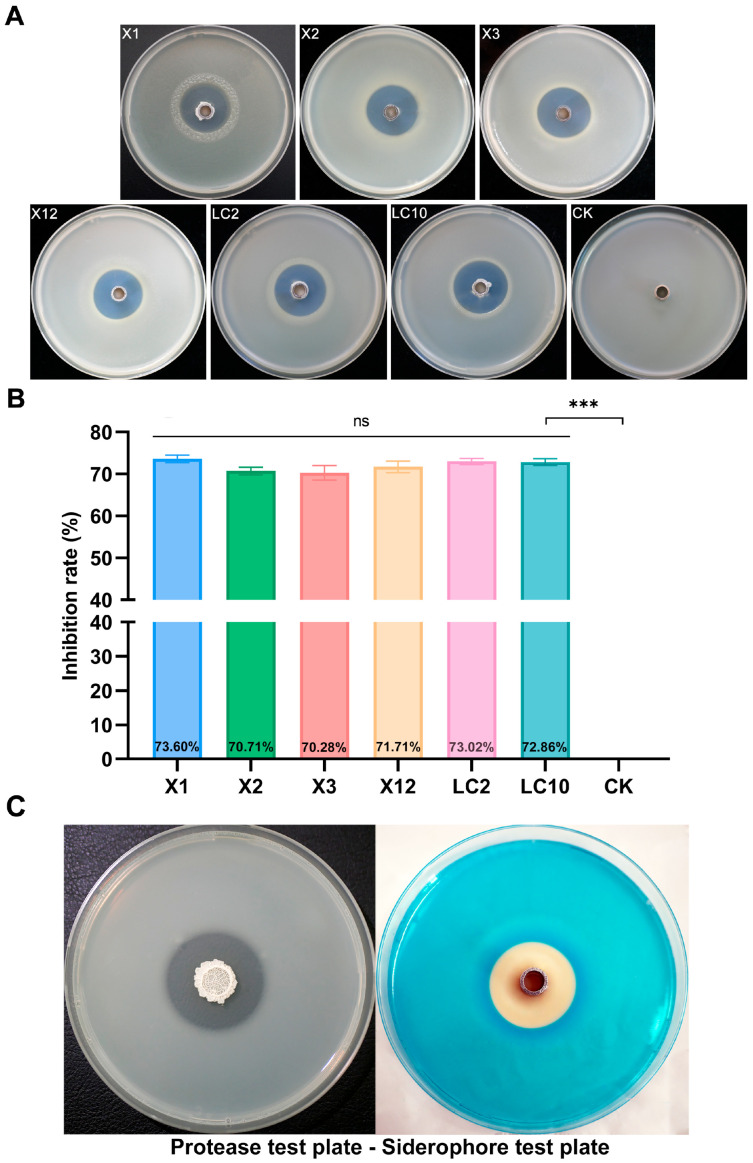
Isolation antimicrobial assay of AN6 against *Xanthomonas oryzae* pv. *oryzae* (*Xoo*). (**A**) *In vitro* antagonistic activity of AN6 against *Xoo* strains. (**B**) Inhibitory rate of AN6 against *Xoo* growth. *** *p* < 0.001; ns, not statistically significant. Statistical analysis was performed using GraphPad software, evaluated through one-way analysis of variance (ANOVA), followed by Dunnett multiple comparison post hoc tests. (**C**) Protease and siderophore production of AN6.

A collection of endophytic bacteria was isolated from rice leaves, and strain AN6 exhibited the strongest antagonistic activity against *Xanthomonas oryzae* pv. *oryzae*, the pathogen responsible for bacterial leaf blight (BLB) of rice, as determined by plate-based antagonism screening (Figure 1A). The inhibition rates of AN6 against six strains of *X. oryzae* pv. *oryzae* (X1, X2, X3, X12, LC2, and LC10) were 73.60%, 70.71%, 70.28%, 71.71%, 73.02%, and 72.86%, respectively (Figure 1B). Furthermore, assays related to plant probiotic traits demonstrated that isolated strain AN6 can secrete proteases and produce siderophores (Figure 1C). However, it lacks cellulase activity and the ability to degrade inorganic phosphorus and organic phosphorus (Supplementary Figure S1). This strain is currently preserved at the China Center for Type Culture Collection (CCTCC) under accession number CCTCC M 20231886.

### 3.2. Morphological and Biological Characteristics of the Isolated Strain AN6

**Figure 2:**
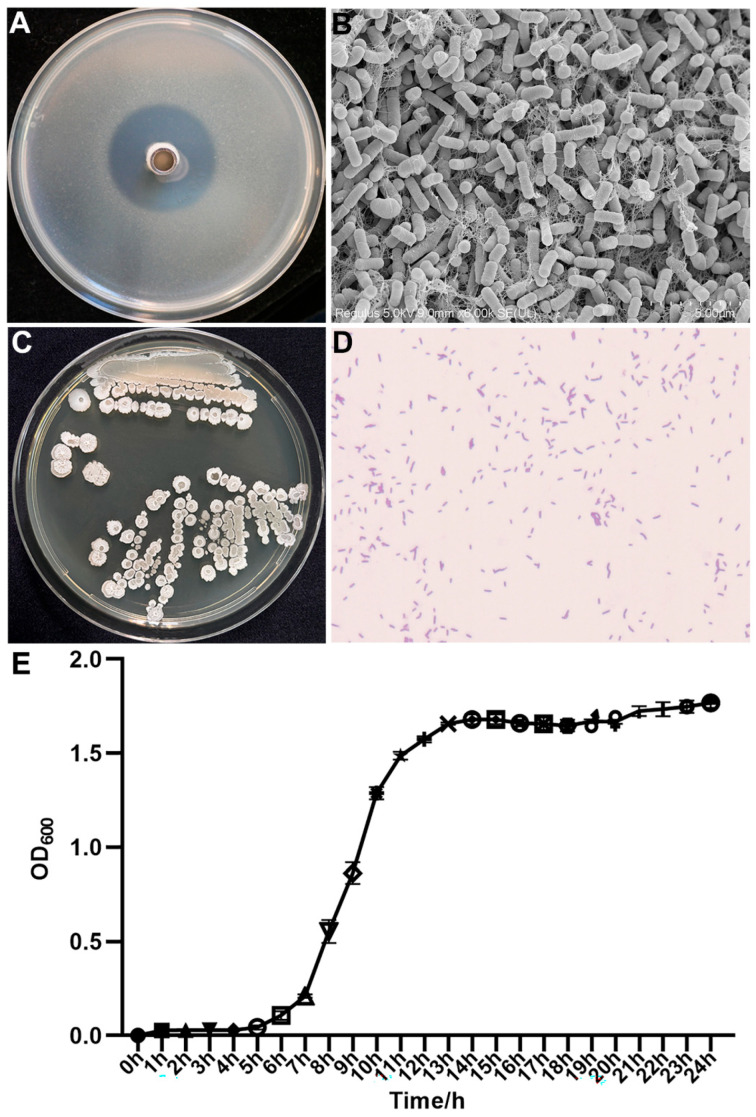
Biological characteristics of AN6 strain. (**A**) Inhibitory effect of AN6 against *Xanthomonas oryzae* pv. *oryzae* (*Xoo*). (**B**) Morphology of the AN6 strain observed under a scanning electron microscope. Scale: 5.00 µm. (**C**) Colony morphology of the AN6 strain on LA medium. (**D**) Morphology of Gram-stained AN6 strain. (**E**) Growth curves of the AN6 strain cultured in LB at 30 °C and 160 rpm. The experiments were repeated three times with 3–5 internal replicates each.

Strain AN6 exhibited the strongest inhibitory effect against *X. oryzae* pv. *oryzae* (*Xoo*) (Figure 2A) and was therefore selected for morphological characterization. The single colony of AN6 on the NA medium showed round, white colonies with a rough surface (Figure 2C). The observation using scanning electron microscopy revealed that AN6 cells were rod-shaped with blunt ends, measuring approximately 1.55 μm × 0.62 μm on average (Figure 2B). Gram staining indicated that AN6 is a Gram-positive bacterium (Figure 2D). Growth curve analysis showed that AN6 exhibited its highest growth rate after 9–10 h of incubation at 30 °C with shaking at 160 rpm. After 13 h, the growth began to plateau, reaching a maximum optical density of approximately OD_600_ ≈ 1.80 (Figure 2E).

### 3.3. Antagonistic Activity of AN6 Strain Cell-Free Supernatant

**Figure 3:**
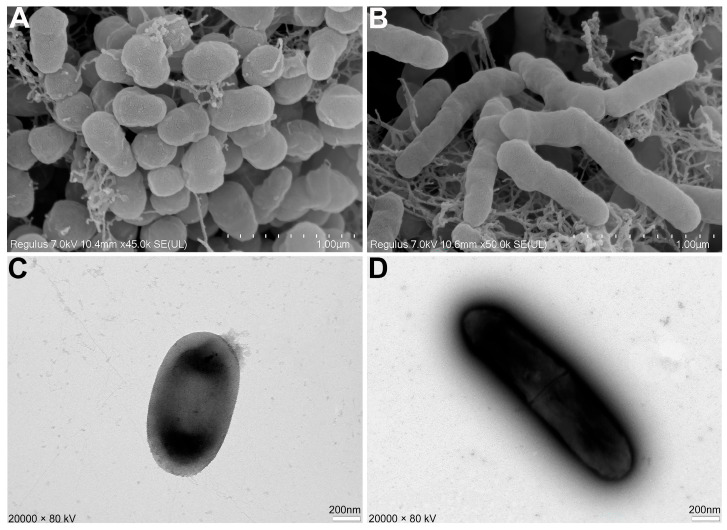
SEM and TEM images of cell morphology of *Xanthomonas oryzae* pv. *oryzae* LC2. (**A**,**B**) SEM image of *Xanthomonas oryzae* pv. *oryzae* LC2 cell morphology under normal conditions and when exposed to AN6 cell-free supernatant, respectively. Scale: 1.00 µm. (**C**,**D**) TEM image of *Xanthomonas oryzae* pv. *oryzae* LC2 cell morphology under normal conditions and when exposed to AN6 cell-free supernatant, respectively. Scale: 200.00 nm.

To further investigate the effect of AN6 on the morphology of *X. oryzae* pv. *oryzae* LC2, scanning electron microscopy (SEM) and transmission electron microscopy (TEM) were used to observe the morphological changes of *Xoo* LC2 cells under both normal culture conditions and stress induced by the sterile cell-free supernatant of *Bacillus velezensis* AN6. SEM analysis revealed that *Xoo* LC2 cells under normal conditions had smooth surfaces and maintained a typical rod-shaped morphology (Figure 3A). In contrast, cells cultured in the presence of AN6 sterile cell-free supernatant exhibited clear morphological abnormalities, including elongation and deformation, indicating membrane stress and structural damage (Figure 3B). The TEM observations supported these findings. *Xoo* LC2 cells grown under normal conditions displayed an intact ultrastructure with clearly defined cell walls and no observable abnormalities (Figure 3C). However, cells treated with the AN6 supernatant showed significant morphological alterations, including indistinct or disrupted cell wall structure and plasmolysis-like separation, suggesting severe physiological stress and, in some cases, cell lysis (Figure 3D). Together, these SEM and TEM results demonstrate that the sterile cell-free supernatant of *Bacillus velezensis* AN6 can significantly damage the cellular structure of *Xoo* LC2, resulting in pronounced morphological abnormalities and physiological dysfunction. These findings provide direct morphological evidence of the antagonistic activity of AN6.

### 3.4. Biocontrol Effect of AN6 Against Xoo

**Figure 4:**
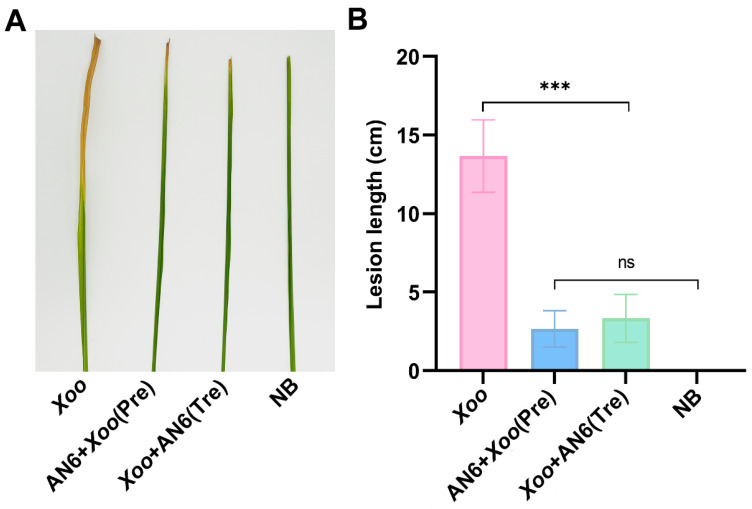
Efficacy of *B. velezensis* AN6 for controlling *Xoo* LC2 in a light incubator. The indoor trials were performed using cultivar YX650. The groups were as follows: *Xoo* LC2 only (Control), NB only, rice leaves sprayed with *B. velezensis* AN6 12 h before inoculation with *Xoo* LC2 suspension (AN6-Pre, prevention strategy), and 12 h after inoculation with *Xoo* LC2 suspension (AN6-Tre, treatment strategy). (**A**) The severity of rice bacterial blight disease (BB) 15 days after *Xoo* LC2 inoculation. (**B**) Lesion length of *Xoo* LC2 growth. *** *p* < 0.001; ns, not statistically significant compared to NB only. Statistical analysis was performed using GraphPad software, evaluated through one-way analysis of variance (ANOVA), followed by Dunnett multiple comparison post hoc tests.

To assess the biocontrol potential of *B. velezensis* AN6 against *X. oryzae* pv. *oryzae* (*Xoo*), a leaf-clipping inoculation assay was conducted on potted rice plants in a light incubator. As shown in Figure 4, leaves in the control group, which were inoculated with *Xoo* alone, developed typical bacterial blight symptoms; the leaves appeared withered and had visible chlorosis, with an average lesion length of 13.67 cm. In the preventive treatment group, the leaves were pretreated with an AN6 bacterial suspension prior to *Xoo* inoculation; the severity of disease was significantly reduced, resulting in an average lesion length of only 2.67 cm. In the curative treatment group, where the AN6 suspension was applied after *Xoo* inoculation, lesion development was also substantially mitigated, with an average lesion length of 3.33 cm. Compared with the control, the AN6-Pre and AN6-Tre treatments dramatically reduced the severity of BB on YX650, with relative control efficiencies of 80.49% and 75.61%, respectively. These findings indicate that *B. velezensis* AN6 exhibits strong *in vitro* biocontrol efficacy against *Xoo*, and there was no significant difference between the AN6-Pre and AN6-Tre groups, which further highlights the practical flexibility of AN6, supporting its potential application as a biological agent for the management of rice bacterial blight.

### 3.5. Antifungal Activity of Strain AN6 In Vitro

**Figure 5:**
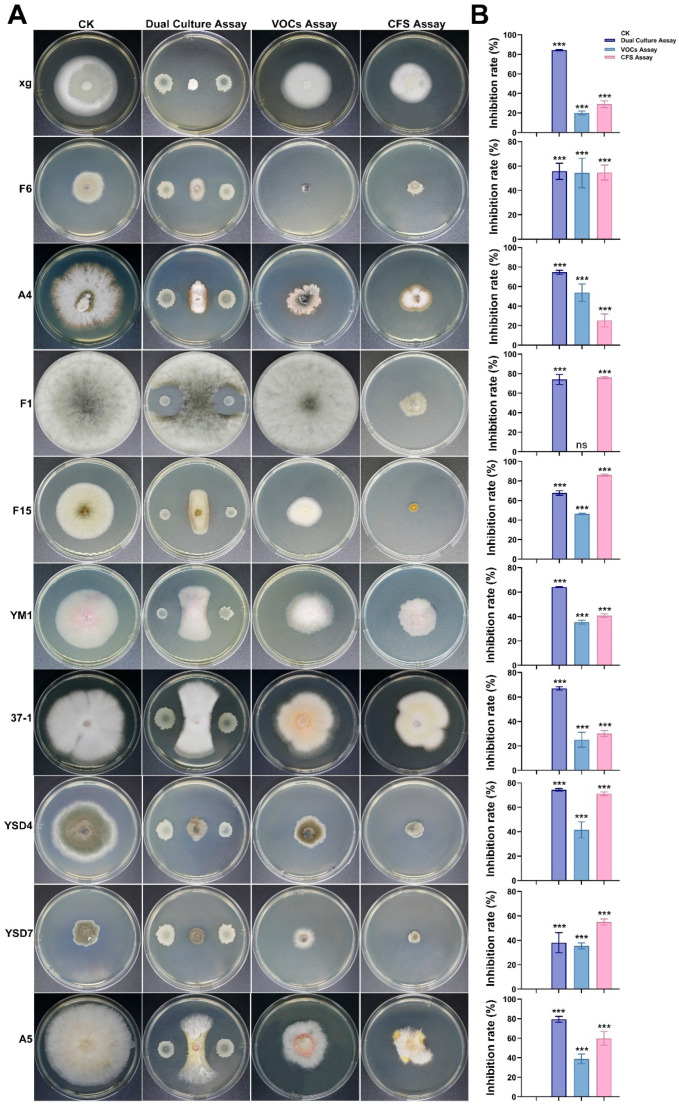

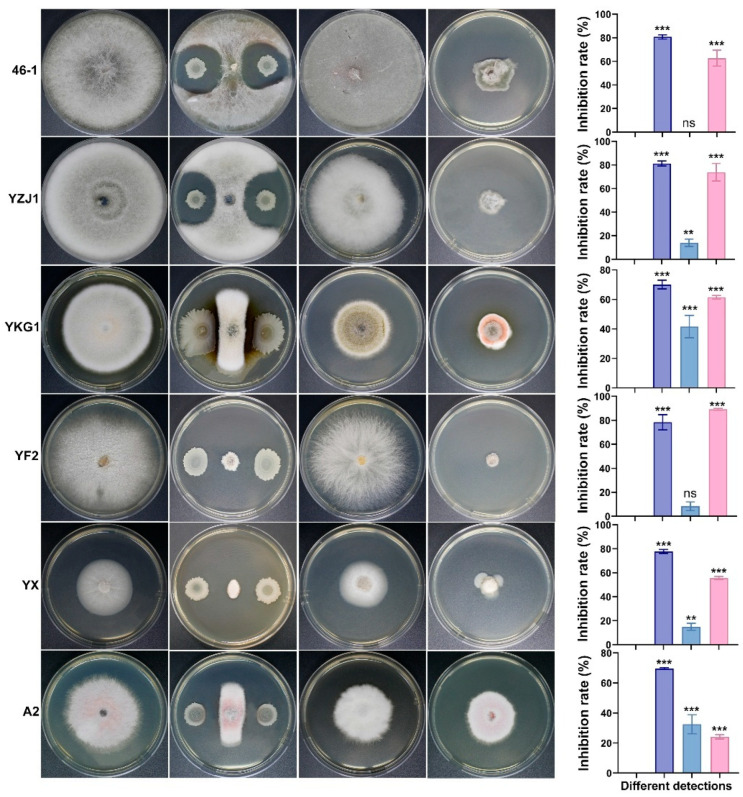
Antifungal activity of AN6 against different fungi. (**A**) *In vitro* inhibition activity of strain AN6, its volatile organic compounds, and cell-free supernatant on mycelial growth of different fungi. CK: Control samples consisting of the fungal plate that was not inoculated with bacteria. Dual-culture assay measures inhibitory effect of AN6 on fungi. VOC assay measures inhibition of fungi by strain AN6 and its VOCs. CFS assay measures inhibition of fungi by aseptic fermentation filtrate of AN6. (**B**) Statistical analysis of AN6’s inhibitory impact on different pathogenic fungi. ** *p* < 0.01; *** *p* < 0.001; ns, not statistically significant vs. CK. Statistical analysis was conducted using GraphPad software: one-way ANOVA followed by Dunnett’s multiple comparison post hoc tests.

To evaluate the broad-spectrum antifungal activity of strain AN6, 16 phytopathogenic fungi were tested (Supplementary Table S1) and three distinct detection methods were employed. The results are shown in Figure 5. The dual-culture assay revealed that AN6 inhibited mycelial growth of these pathogens by 38.09% to 84.24%. Compared with the control group, AN6 exhibited an inhibitory activity of over 80% against *M. oryzae* xg, *N. oryzae* 46-1, and *N. oryzae* YZJ1. The maximum mycelial growth inhibition percentage was against *M. oryzae* xg (84.24 ± 1.05%), followed by *N. oryzae* YZJ1 (81.24 ± 2.10%). The minimum inhibition percentage was against *B. cynodontis* YSD7 (38.09 ± 2.82%), followed by *A. tenuissima* F6 (55.79 ± 2.27%) and *F. oxysporum* YM1 (64.07 ± 0.37%). The VOC assay showed that the inhibition rate of the VOCs produced by AN6 on the mycelial growth of pathogens ranged from 0 to 54.44%. The maximum mycelial growth inhibition percentage was against *A. tenuissima* F6 (54.44 ± 2.71%), followed by *E. latusicollum* A4 (53.21 ± 6.00%). The inhibition rate of the VOCs produced by AN6 against *L. theobromae* F1 and *N. oryzae* 46-1 mycelia was almost zero. The CFS assay showed that the inhibition rate of AN6 and its aseptic fermentation filtrate on the mycelial growth of the tested pathogens ranged from 25.46% to 89.17%. Compared with the control group, AN6 had an inhibitory activity of more than 80% against *A. alternata* F15 and *N. sphaerica* YF2. The maximum mycelial growth inhibition percentage was against *N. sphaerica* YF2 (89.17 ± 0.72%), followed by *A. alternata* F15 (86.27 ± 0.98%). The minimum inhibition percentage was against *E. latusicollum* A4 (25.26 ± 6.52%), followed by *M. oryzae* xg (29.09 ± 3.15%) and *F. fujikuroi* 37-1 (30.18 ± 2.34%). The results of the dual-culture, VOC, and CFS assays showed that AN6 had inhibitory effects on the 16 pathogenic fungi to varying degrees. Notably, AN6 displayed relatively strong inhibitory effects in the dual-culture and CFS assays, with maximum inhibition rates of 84.24% and 89.17%, respectively. These findings suggest that strain AN6 possesses substantial inhibitory potential against the 16 tested pathogenic fungi, highlighting its promise as a biocontrol candidate.

### 3.6. Complete Genome Sequence and Biosynthesis Gene Clusters of Strain AN6

**Figure 6:**
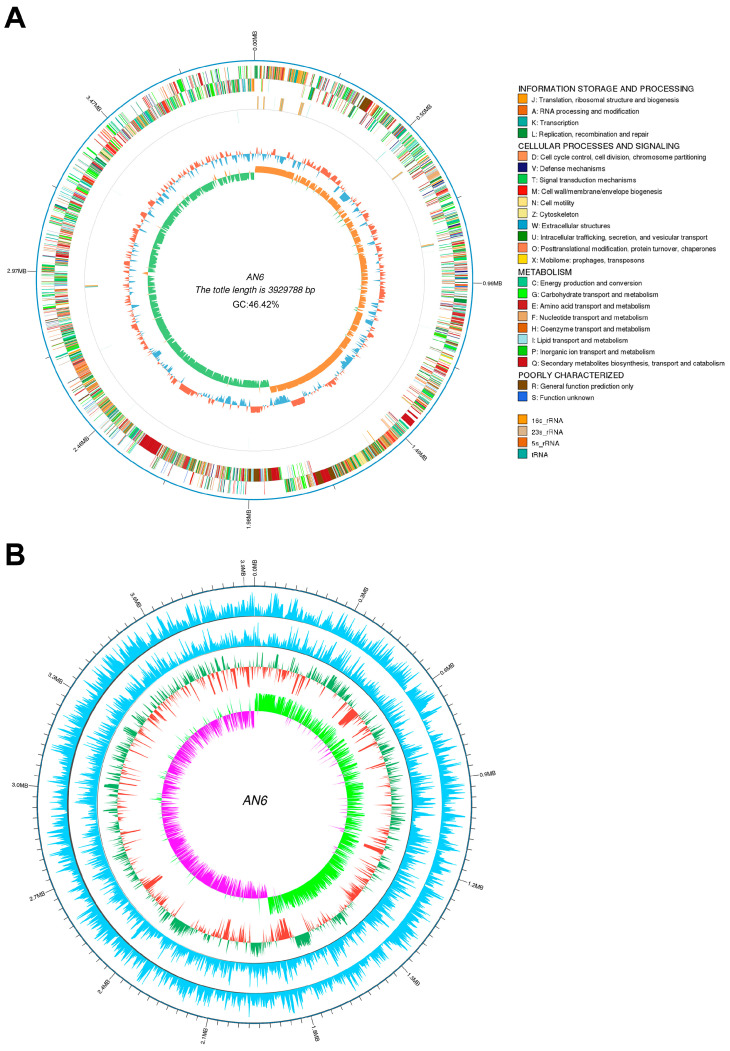
The complete genome map and epigenetic modification distribution map of *Bacillus velezensis* AN6. (**A**) Circular genome map of *Bacillus velezensis* AN6 visualized using Circos 0.69-9 software. From the outermost to the innermost ring: ring 1 displays the genome coordinates and physical positions along the chromosome (black line); ring 2 shows protein-coding genes located on the forward and reverse strands; ring 3 indicates non-coding RNAs (ncRNAs); ring 4 presents the GC content; ring 5 shows the GC skew, with positive skew shown in orange and negative skew shown in green. (**B**) Whole-genome methylation map of *Bacillus velezensis* AN6. The concentric rings in the figure represent, from the outside to the inside, genomic coordinates; the distribution of modification sites on the forward (sense) strand, calculated using a 2000 bp window and a 2000 bp step size; the distribution of modification sites on the reverse (antisense) strand using the same parameters; the outer two blue rings represent the distribution of modification sites on the sense strand (using a 2000 bp window with a 2000 bp step) and on the antisense strand (using a 2000 bp window with a 2000 bp step), respectively; the GC content, also calculated with a 2000 bp window and step size (red segments pointing inward indicate regions with a GC content lower than the genome-wide average, while green segments pointing outward indicate regions with a higher GC content and the taller the peak, the greater the deviation from the average); and the innermost ring represents the GC skew, calculated using the formula (G − C)/(G + C), with a 2000 bp window and step size (pink segments pointing inward indicate regions where the G content is lower than the C content, while light green segments pointing outward indicate the opposite).

**Figure 7:**
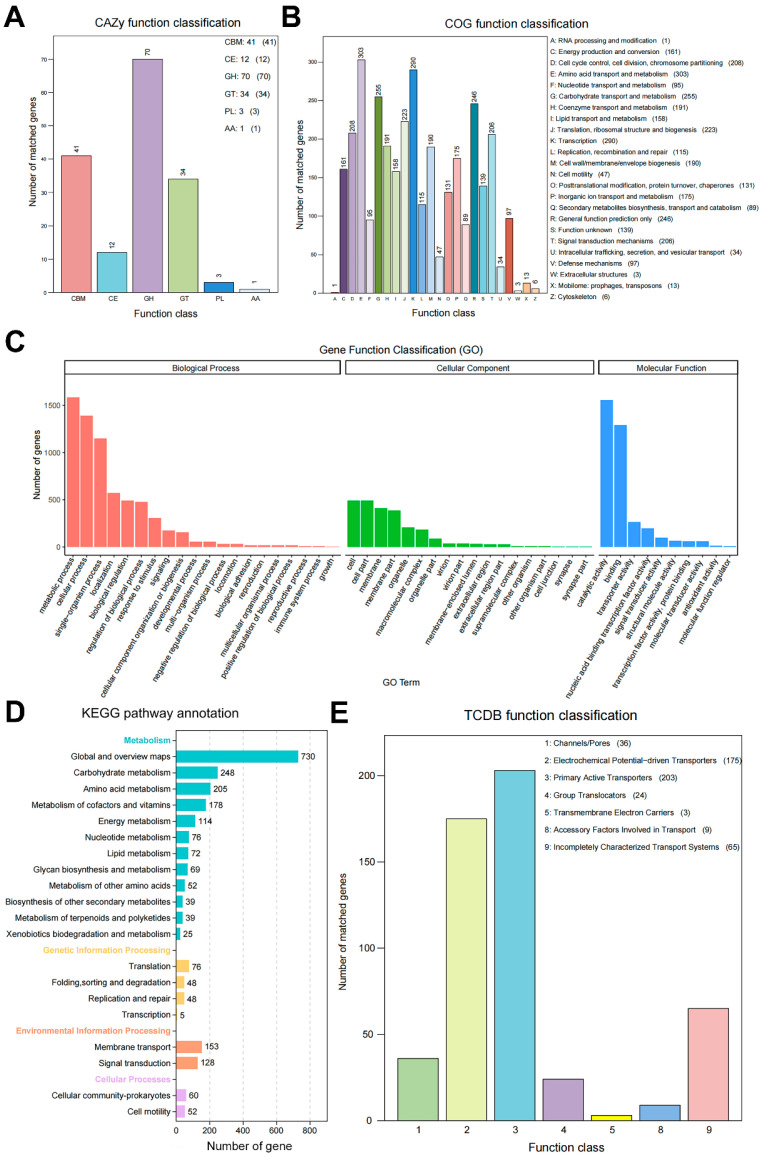
The functional annotation and gene prediction results for the strain AN6. (**A**) CAZy (Carbohydrate-Active enZYmes Database) functional classification. (**B**) COG (clusters of orthologous groups) functional classification. (**C**) Gene distribution based on Gene Ontology. (**D**) KEGG (Kyoto Encyclopedia of Genes and Genomes) metabolic pathways annotation with the gene functions. (**E**) TCDB (Transporter Classification Database) functional classification.

A total of 901,111 long reads were generated using PacBio sequencing, yielding 6,130,597,881 bases with an average read length of 6803 bp and an N50 of 9742 bp. These high-quality reads were used to assemble the genome of strain AN6. The final assembly resulted in a complete circular chromosome with 3,929,788 bp and a GC content of 46.50%. The circular chromosome map of AN6 is presented in Figure 6A. The assembled genome has been deposited in the NCBI database under accession number JBKKFY000000000. A genome-wide DNA methylation analysis of AN6 was performed and visualized as a circular map (Figure 6B). The AN6 genome was found to contain 2344 m4C modification sites, 1154 m5C sites, and 116 m6A sites. Genome annotation predicted 4025 protein-coding genes, 86 tRNAs, 27 rRNAs, 9 CRISPR arrays, 9 genomic islands, and 13 prophage regions. All the predicted protein sequences of AN6 were compared to those in the Nr, COG, GO, KEGG, TCDB, CAZy, Swiss-Prot and Pfam databases; the annotation summary is presented in Table 2. There are 3939 proteins in AN6 were classified into 20 Nr species (Table S2.1). A total of 2993 proteins in AN6 were classified into 24 COG families (Figure 7B, Table S2.2) and a total of 2714 genes were annotated with a GO function, which are categorized as biological process, cellular component, or molecular function (Figure 7C, Table S2.3). A total of 3875 genes were annotated using the KEGG database, which were distributed across 20 pathways in four major functional categories (Figure 7D, Table S2.4). Protein domains were identified in 2714 proteins (Table S2.5). A total of 3302 proteins were annotated using the Swiss-Prot database (Table S2.6). A total of 3875 genes were annotated using the TCDB database, which were distributed across seven major functional categories (Figure 7E, Table S2.7). A total of 156 genes had CAZy-annotated functions covering six major functional categories (Figure 7A, Table S2.8).

### 3.7. Comparative Genomics Analysis

**Figure 8:**
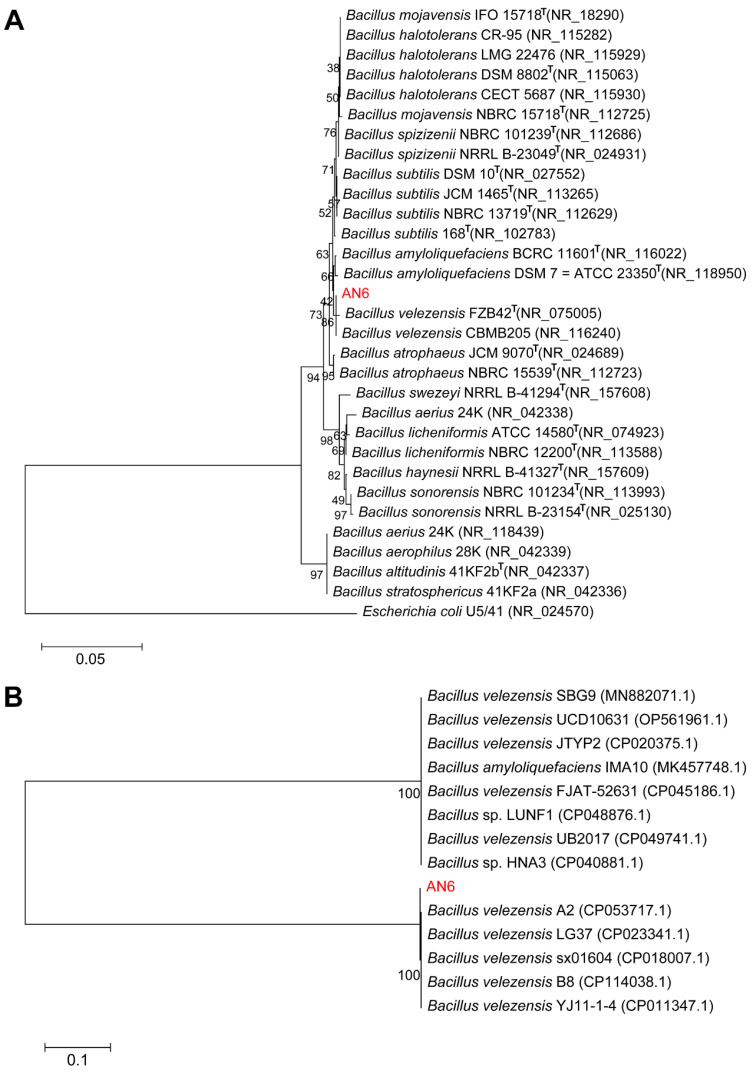
Phylogenetic analysis of AN6 and *Bacillus* strains. (**A**) Neighbor-joining phylogenetic tree showing the phylogenetic relationship of strain AN6 with other members of the *Bacillus* genus based on 16S rRNA gene sequences. The gene sequences of *E. coli* U5/41 (NR_024570) were utilized as the root for constructing the phylogenetic tree. The bootstrap consensus was inferred from 1000 replicates. Scale bar: 0.01 substitutions per nucleotide position. (**B**) Neighbor-joining phylogenetic tree showing the phylogenetic relationship of strain AN6 with other members of the *Bacillus* genus based on *gyrA* gene sequences. The bootstrap consensus was inferred from 1000 replicates. Scale bar: 0.01 substitutions per nucleotide position. The strain obtained in this study is highlighted in red.

The 16S rRNA and *gyrA* genes of strain AN6 were amplified by PCR and subsequently sequenced. BLAST analysis revealed that the 16S rRNA gene sequence of AN6 shared the highest similarity with that of *B. velezensis* CBMB205 (NR_116240) and *B. velezensis* FZB42 (NR_075005), with sequence identities of 99.93% and 99.86%, respectively. Based on these results, a phylogenetic tree was constructed using the maximum likelihood method. The resulting tree showed that AN6 clustered within the same evolutionary branch as *B. velezensis* FZB42 and CBMB205 (Figure 8A). To further resolve the phylogenetic placement of AN6 among *Bacillus* species, phylogenetic analysis was performed based on the *gyrA* gene. As shown in Figure 8B, AN6 was most closely related to *B. velezensis* strains A2, LG37, sx01604, B8, and YJ11-1-4. These combined results clearly support the classification of strain AN6 as a member of *Bacillus velezensis*.

**Figure 9:**
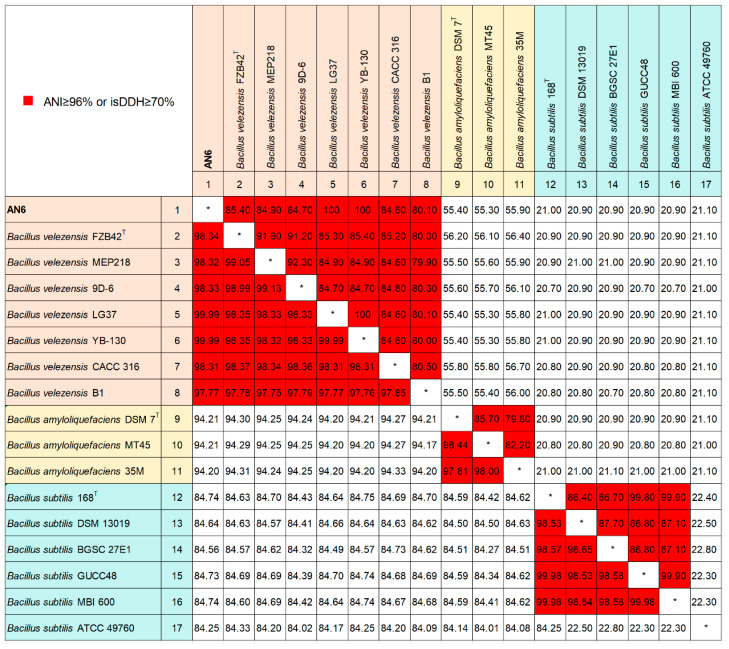
Pairwise average nucleotide identity (ANI) and in silico DNA–DNA hybridization (*is*DDH) values derived from the analysis of the whole genomes of AN6 and 16 other *Bacillus* strains. The left lower triangle illustrates the ANI values, whereas the right upper triangle displays the *is*DDH values. Squares highlighted in red indicate an ANI ≥ 96% or *is*DDH ≥ 70%. The superscript T on the right denotes the standard strain. The asterisk (*) marks self-vs-self comparisons, which are omitted as they are non-informative.

Furthermore, average nucleotide identity (ANI) and in silico DNA–DNA hybridization (*is*DDH) analyses were conducted between strain AN6 and 16 publicly available *Bacillus* strain genomes. Based on the established species delineation thresholds of ANI ≥ 96% and *is*DDH ≥ 70%, the results (Figure 9) were consistent with the phylogenetic relationships observed in Figure 7. Strain AN6 exhibited ANI values of 98.34%, 98.32%, 98.33%, 99.99%, 99.99%, 98.31%, and 97.77% with *B. velezensis* strains FZB42^T^ (type strain), MEP218, 9D-6, LG37, YB-130, CACC 316, and B1, respectively. The corresponding *is*DDH values were 85.40%, 84.90%, 84.70%, 100%, 100%, 84.60%, and 80.10%. All values exceeded the established thresholds for species-level classification, further confirming that strain AN6 is a strain of *Bacillus velezensis*.

**Figure 10:**
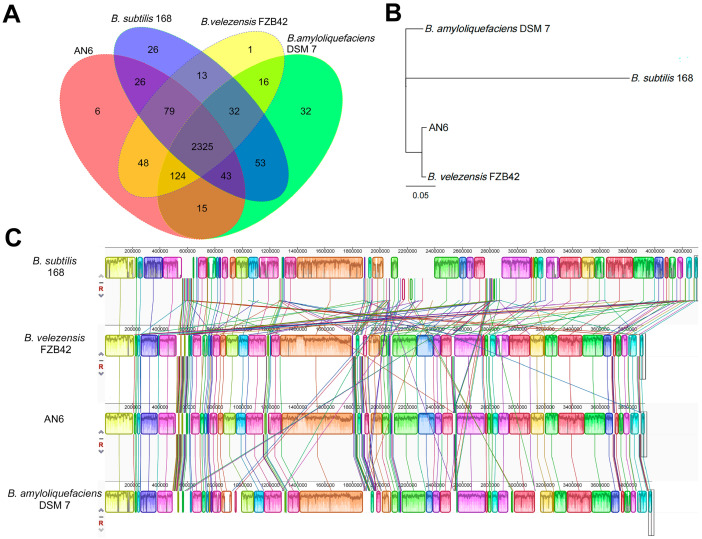
Comparative analysis of the genomes of AN6, *B. velezensis* FZB42, *B. amyloliquefaciens* DSM7, and *B. subtilis* 168. (**A**) Venn diagram showing ortholog gene families between AN6 and *B. velezensis* FZB42, *B. amyloliquefaciens* DSM7, and *B. subtilis* 168. (**B**) Analysis of the evolutionary relationship. The bootstrap consensus was inferred from 1000 replicates. Scale bar: 0.01 substitutions per nucleotide position. (**C**) Collinearity analysis. Boxes of similar colors represent syntenic regions, boxes below the horizontal line represent inversion regions, and rearrangements are represented by colored lines.

Gene family clustering analysis was performed on *B. velezensis* AN6, *B. velezensis* FZB42, *B. subtilis* 168, and *B. amyloliquefaciens* DSM 7. The results showed that strain AN6 contained 2666 gene families, including 6 unique gene families. *B. velezensis* FZB42, *B. subtilis* 168, and *B. amyloliquefaciens* DSM 7 possessed 2638, 2597, and 2640 gene families, with 1, 26, and 31 unique gene families, respectively. A total of 2325 gene families were conserved across all four strains, accounting for 87.21%, 88.13%, 89.53%, and 88.07% of the total gene families in AN6, FZB42, 168, and DSM 7, respectively (Figure 10A). Based on the single-copy orthologous genes identified through gene family clustering, multiple-protein sequence alignment was performed using MUSCLE, and a phylogenetic tree was constructed using the maximum likelihood method in PHYML. The phylogenetic tree showed that AN6 clustered closely with *B. velezensis* FZB42, indicating a close evolutionary relationship between the two strains (Figure 10B). Whole-genome synteny analysis between AN6 and *B. velezensis* FZB42, *B. subtilis* 168, and *B. amyloliquefaciens* DSM 7 revealed a high degree of gene homology and overall collinearity, particularly between AN6 and *B. velezensis* FZB42. Nevertheless, several genomic rearrangements were observed, including gene inversions, transpositions, translocations, and deletions (Figure 10C). These findings suggest that while the core genome is largely conserved across these *Bacillus* species, structural variations have contributed to their genomic divergence.

Secondary metabolite biosynthetic gene clusters in the AN6 genome were predicted using antiSMASH v8.0.4. The analysis revealed a total of 13 biosynthetic gene clusters (Table 3), including those responsible for the nonribosomal synthesis of lipopeptides (surfactin, fengycin, and bacillibactin), polyketides (difficidin, bacillaene, and macrolactin H), and a dipeptide antibiotic (bacilysin). In addition, six gene clusters were associated with the biosynthesis of unknown terpenes, polyketides, and class II lanthipeptides.

## 4. Discussion

Rice is one of the most important staple crops worldwide, and various pathogens such as *X. oryzae* pv. *oryzae* [56], *M. oryzae* [57], *N. oryzae* [58], *Nigrospora* spp. [59], *Epicoccum* spp. [60], *Bipolaris* spp. [61], *Alternaria* spp. [62], and *Fusarium* spp. [63] are responsible for major constraints on its cultivation and productivity. Plant diseases caused by phytopathogenic bacteria and fungi seriously threaten global rice production, leading to significant yield and economic losses. Biocontrol strategies provide environmentally friendly solutions to manage plant diseases, decreasing the dependence on harmful chemical treatments. In recent decades, biological control has emerged as a promising and eco-friendly strategy for plant disease management due to its high efficiency, broad-spectrum antagonistic effects, and sustainability compared to chemical pesticides [64,65]. Previous studies have demonstrated that beneficial microorganisms such as *Bacillus*, *Pseudomonas*, and *Streptomyces* can suppress diverse plant diseases through mechanisms including antibiotic production, secretion of lytic enzymes, and induction of systemic resistance [66]. In the present study, we successfully isolated a novel *Bacillus velezensis* strain AN6 from rice plants, which exhibited strong antagonistic activity against *X. oryzae* pv. *oryzae* as well as 16 other fungi *in vitro*. Therefore, the complete genome of *B. velezensis* AN6 was sequenced for detailed analysis.

Numerous studies have reported that *B. velezensis* strains, as novel biocontrol agents, exhibit significant potential in the management of various plant diseases [67,68]. In addition, the use of *B. velezensis* in biocontrol research has become increasingly widespread. In particular, *B. velezensis* strain CC09 has been demonstrated to effectively control wheat powdery mildew [69], *B. velezensis* AL7 possesses biocontrol potential against cotton Verticillium wilt [70], *B. velezensis* SF334 exhibits significant antagonistic activity against rubber tree leaf anthracnose [71], *B. velezensis* BRI3 can effectively antagonize *Sclerotinia sclerotiorum* (Lib.) [72], and *B. velezensis* Y6 has been shown to suppress rice sheath blight caused by *R. solani* [73]. These findings offer a more environmentally friendly method for managing diseases. In our study, strain AN6 exhibited strong inhibitory activity against *X. oryzae* pv. *oryzae* (Figure 1), significantly suppressing *Xoo* growth (Figure 3) and demonstrating effective biocontrol in pot assays (Figure 4). Its CFS caused severe morphological damage to *Xoo*, likely mediated by lipopeptides, polyketides, or other extracellular metabolites, as supported by genome-encoded secondary metabolite biosynthetic gene clusters (Table 2). This is consistent with previous studies [41], where the CFS of strain Bv-303 significantly inhibited *Xoo* growth *in vitro*, and *in vivo* applications of the cell-culture broth (CCB), CFS, or cell-suspension water (CSW) enhanced the resistance of rice plants to bacterial blight (BB). To further elucidate the key active compounds, future work could combine LC–MS/MS metabolite profiling, bioassay-guided fractionation, and targeted gene-cluster validation. The dual-culture assay can assess direct antagonism, the volatile organic compound assay can assess indirect antagonism, and the sterile fermentation filtrate assay can assess the ability of the strain to produce antimicrobial metabolites. The results showed that AN6 exhibited antimicrobial activity in all three assays, but the inhibitory activity against different fungal pathogens varied between the assays. The dual-culture assay and the sterile fermentation filtrate assay demonstrated the strongest inhibitory activity, with the highest inhibition rates reaching 84.24 ± 1.05% and 89.17 ± 0.72%, respectively. Comparing these two methods, we found that for most fungal pathogens, AN6 exhibited the strongest antifungal activity in the dual-culture assay. For fast-growing fungal pathogens (*L. theobromae* F1, *N. oryzae* 46-1, and *N. oryzae* YZJ1), the dual-culture assay showed only moderate inhibition, whereas the sterile fermentation filtrate assay demonstrated stronger inhibition (Figure 5). This result supports the possibility that, for certain fungal pathogens, direct cell-to-cell interaction and contact-dependent induction of antimicrobial metabolites may be more important than constitutively secreted soluble compounds. Similar “interaction-dependent induction” of lipopeptides and other antifungal secondary metabolites has been reported in antagonistic *Bacillus* strains. The presence of *Fusarium oxysporum* f. sp. *strigae* (Fos) in dual-plate cultures led to an increase in bacillomycin D production by *Bacillus* strains [74]. This suggests that, for different fungal pathogens, targeted control strategies may be implemented based on the specific antagonistic mechanisms of the biocontrol agent. Compared to some microbial strains that have shown potential in inhibiting *M. oryzae* in many studies in dual-culture assays, the efficacy of AN6 against *M. oryzae* is equivalent to or slightly higher. For example, Wang et al. reported that *B. velezensis* LJBV19 showed significant inhibitory effects on the growth of *M. oryzae* with an inhibition ratio of 75.55%, while Wockenfuss et al. reported that *B. velezensis S4* significantly inhibited the growth of *M. oryzae* by 38.53% [75,76]. Similarly, compared with the inhibitory effect of *B. velezensis* B31 on *N. oryzae* [77], the inhibitory effect of AN6 was relatively higher. It has been reported that different *Bacillus* strains have inhibitory effects on different *Fusarium* species [78,79,80]. This study found that AN6 effectively inhibits at least five *Fusarium* species. *B. oryzae* is the main pathogen that causes brown spot disease of rice (BSR) [81]. Strain AN6 exhibited similar *in vitro* inhibitory activity against *B. oryzae* as *B. velezensis* LS123N [82]. *N. oryzae* is the pathogen that causes rice stem rot [58] and *B. cynodontis* is the fungus responsible for leaf blight [83,84]. To our knowledge, this is the first report of the antagonistic activity of *B. velezensis* against *B. cynodontis* and *N. oryzae*.

Numerous studies have shown that *B. velezensis* produces VOCs with strong inhibitory effects on bacterial and plant pathogen growth, such as the VOCs from *B. velezensis* ZSY-1, which significantly suppressed six types of plant pathogenic fungi [85], and the VOCs released by *B. velezensis* T9, which inhibited the growth of Apiospora mold on sugarcane caused by *A. arundinis* [86]. The CFS of *B. velezensis* exhibited significant antifungal effects by inhibiting spore germination. The CFS of *B. velezensis* exhibited potent antifungal activity against *B. cinerea* and *P. olsonii in vitro*. It hindered spore germination, germ tube elongation, and hyphal growth, and damaged mycelial cells by triggering excessive ROS accumulation [87,88].

The phylogenetic tree analysis using 16S rRNA gene sequences showed that the AN6 strain clusters closely with *B. velezensis* FZB42 and *B. velezensis* CBMB205 (Figure 8A). The phylogenetic tree analysis using *gyrA* gene sequences showed that the AN6 strain clusters closely with *B. velezensis* A2, *B. velezensis* LG37, *B. velezensis* sx01604, *B. velezensis* B8, and *B. velezensis* YJ11-1-4 (Figure 8B), indicating a close relationship between AN6 and these strains. The ANI and *is*DDH analyses revealed that AN6 could be classified as *B. velezensis* due to the high degree of genomic homogeneity between AN6 and *B. velezensis* (Figure 9). Gene family clustering revealed that AN6 shares 2325 gene families with *B. velezensis* FZB42, *B. subtilis* 168, and *B. amyloliquefaciens* DSM 7, and contains 6 unique gene families (Figure 10A). The phylogenetic analysis based on single-copy orthologous genes indicated that strain AN6 and *B. velezensis* FZB42 belong to the same evolutionary lineage (Figure 10B). The whole-genome collinearity analysis of AN6, *B. velezensis* FZB42, *B. subtilis* 168, and *B. amyloliquefaciens* DSM 7 demonstrated a high degree of gene homology and overall collinearity, with the similarity between AN6 and *B. velezensis* FZB42 being particularly pronounced (Figure 10C). However, several genomic rearrangements were also observed, including inversions, transpositions, translocations, and deletions.

Several studies indicated that the effectiveness of *Bacillus* strains as biocontrol agents relies on both their ability to colonize target tissues and the presence of a complete, functional biosynthetic gene cluster for producing and secreting secondary metabolites such as iturin, bacillomycin, fengycin, and surfactin [89,90,91,92]. Genome mining identified a total of thirteen secondary metabolite biosynthesis gene clusters in the genome of strain AN6. Seven clusters showed significant similarity (similarity ≥ 75%) to known BGCs, including those for the synthesis of surfactin, macrolactin H, bacillaene, fengycin, difficidin, bacillibactin, and bacilysin (Table 3). Surfactin is a natural surfactant that is primarily produced by *Bacillus* species and exhibits antibacterial properties. Macrolactin H is a member of a class of macrolide natural products that generally exhibits multiple biological activities, including antibacterial, antifungal, antiviral, antitumor, and anti-inflammatory activities [93]. Bacillaene is a linear polyketide/nonribosomal peptide produced by *Bacillus* species through the activity of trans-acyltransferase polyketide synthetase [94]. Fengycin is a cyclic lipopeptide antibiotic that is primarily produced by *Bacillus* species and has demonstrated significant antifungal, antitumor, and antiadhesion properties; the antifungal mechanisms of fengycin mainly involve disruption of cell wall integrity, damage to cell membranes, interference with intracellular metabolism, induction of programmed cell death and autophagy, and activation of plant defense responses [95]. Fengycin is likely a major contributor to the antifungal activity of AN6. Difficidin is a polyketide antibiotic produced by *Bacillus* species via the trans-acyltransferase polyketide synthase pathway [96]. Its mode of action involves inhibition of bacterial protein synthesis and disruption of key cellular metabolic processes. Previous studies have shown that difficidin exhibits potent bactericidal activity against both Gram-positive and Gram-negative pathogens, including plant-pathogenic *Xanthomonas* spp. and *Pseudomonas* spp., and can cause cell-wall and membrane destruction and suppress the expression of bacterial virulence genes [97]. Therefore, we preliminarily infer that difficidin is likely one of the major contributors to the antibacterial activity of AN6. Bacillibactin is a microbial siderophore produced by *Bacillus* species [98]. Bacilysin is a dipeptide antibiotic made up of L-alanine and L-anticapsin and is produced by certain strains of *Bacillus* species. It is attracting growing interest in industrial agriculture and the pharmaceutical industry due to its strong antagonistic effects on various bacterial, fungal, and algal pathogens [99]. Notably, six of AN6’s BGCs showed minimal similarity to known clusters, indicating that strain AN6 contains genes with potentially novel functions, underscoring its significant research potential.

Rice diseases such as bacterial blight caused by *X. oryzae* pv. *oryzae* and rice blast caused by *M. oryzae* remain major threats to rice production. Although chemical fungicides are widely used, sustainable agriculture requires alternative strategies. In this study, strain AN6 exhibited strong antibacterial activity against *X. oryzae* pv. *oryzae*, causing severe cell deformation *in vitro* and significantly reducing disease severity in planta. Furthermore, AN6 displayed antagonistic activity against *M. oryzae* and other fungal pathogens in plate confrontation, VOC, and soluble metabolite assays. This work highlights a methodological advance by integrating three evaluation approaches within one framework, enabling a more comprehensive assessment of biocontrol potential. Such an integrative strategy overcomes the limitations of single-method studies and better reflects natural microbial interactions. Mechanistically, AN6 suppresses pathogens through multiple complementary strategies, including direct cellular contact, secreted extracellular metabolites, and VOCs, with the inhibitory effects varying depending on the pathogen and assay. Genome analysis revealed 13 secondary metabolite biosynthetic gene clusters, including high-confidence clusters for difficidin, fengycin, and surfactin, which likely underlie the observed antibacterial and antifungal activities. Together, the genomic and phenotypic analyses highlight the multifaceted antagonistic potential of AN6. To our knowledge, this study is the first to demonstrate the biocontrol potential of *B. velezensis* against *Bipolaris cynodontis* and *Nakataea oryzae*, expanding the known spectrum of target pathogens. The AN6 strain shows significant potential as a biocontrol agent for the prevention and control of plant diseases.

## 5. Conclusions

In summary, *B. velezensis* strain AN6 isolated from rice plants exhibited strong biocontrol activity against *X. oryzae* pv. *oryzae* and inhibited multiple phytopathogenic fungi. AN6 can produce proteases, siderophores, antimicrobial secondary metabolites, and VOCs. The whole-genome analysis and secondary metabolite gene cluster prediction provided important clues for future studies on the regulatory mechanisms of its antimicrobial metabolites. To our knowledge, this is the first report demonstrating the biocontrol potential of *B. velezensis* against *Bipolaris cynodontis* and *Nakataea oryzae*. Overall, AN6 shows considerable promise as a biocontrol agent for plant disease management and represents a valuable microbial resource for discovering and exploiting novel antimicrobial compounds.

## Figures and Tables

**Table 1:** Methods for assessing the biological control effect of AN6 on *Xoo*.

Treatment	Experimental Variables		Timing of Biocontrol Agent Addition
	Pathogen	Biocontrol Agent	
Control	*Xoo*	NB	12 h after pathogen inoculation
Pre-treated	*Xoo*	AN6	12 h before pathogen inoculation
Co-treated	*Xoo*	AN6	12 h after pathogen inoculation
NB	NB	NB	12 h after pathogen inoculation

**Table 2:** Genome characteristics of strain AN6.

Database	Number of Annotated Functional Proteins	Proportion/%
Nr	3939	97.86
GO	2714	67.43
KEGG	3875	96.27
COG	2993	74.36
Pfam	2714	67.43
Swiss-Prot	3302	82.04
TCDB	515	12.80
CAZy	156	3.88

**Table 3:** Secondary metabolite clusters in strain AN6 predicted by antiSMASH v8.0.4.

Gene Cluster	Secondary Metabolite(s)	Genome Location	Most Similar Known Cluster	Similarity Confidence
Cluster 1	NRPS	322,200–387,607	Surfactin	High
Cluster 2	PKS-like	924,057–965,301		
Cluster 3	Terpene	1,047,341–1,068,081		
Cluster 4	Lanthipeptide class ii	1,188,574–1,217,462		
Cluster 5	TransAT-PKS	1,384,025–1,472,258	Macrolactin H	High
Cluster 6	TransAT-PKS, T3PKS, and NRPS	1,690,949–1,801,042	Bacillaene	High
Cluster 7	NRPS, transAT-PKS, and betalactone	1,865,673–2,003,474	Fengycin	High
Cluster 8	Terpene	2,028,701–2,050,584		
Cluster 9	T3PKS	2,113,902–2,155,002		
Cluster 10	TransAT-PKS	2,269,988–2,376,170	Difficidin	High
Cluster 11	Terpene precursor	2,399,464–2,420,354		
Cluster 12	NRP metallophore, NRPS, and RiPP-like	3,000,874–3,052,665	Bacillibactin	High
Cluster 13	Other	3,588,975–3,630,393	Bacilysin	High

## Data Availability

Sequence data presented in this study are available in GenBank at https://www.ncbi.nlm.nih.gov/datasets/genome/ (accessed on 30 November 2024) with under the accession numbers listed in the Results Section. Other relevant data are included in the article/Supplementary Materials.

## Supplementary Materials

The following supporting information can be downloaded at: https://www.mdpi.com/article/10.3390/microorganisms13122701/s1, Figure S1: Experimental results for AN6 on the degradation of cellulose, organic phosphorus solubilization, and inorganic phosphorus solubilization; Table S1: Phytopathogenic fungi used in this study (for the *in vitro* antifungal activity analyses of strain AN6); Table S2: The annotation results of the coding genes in the genome of strain AN6.
