# Supplementary figures and images for: Isolation and Genome-Based Characterization of Bacillus velezensis AN6 for Its Biocontrol Potential Against Multiple Plant Pathogens
Source: Microorganisms. 2025 Nov 27;13(12):2701. doi: 10.3390/microorganisms13122701 (PMC12735488; doi:10.3390/microorganisms13122701)

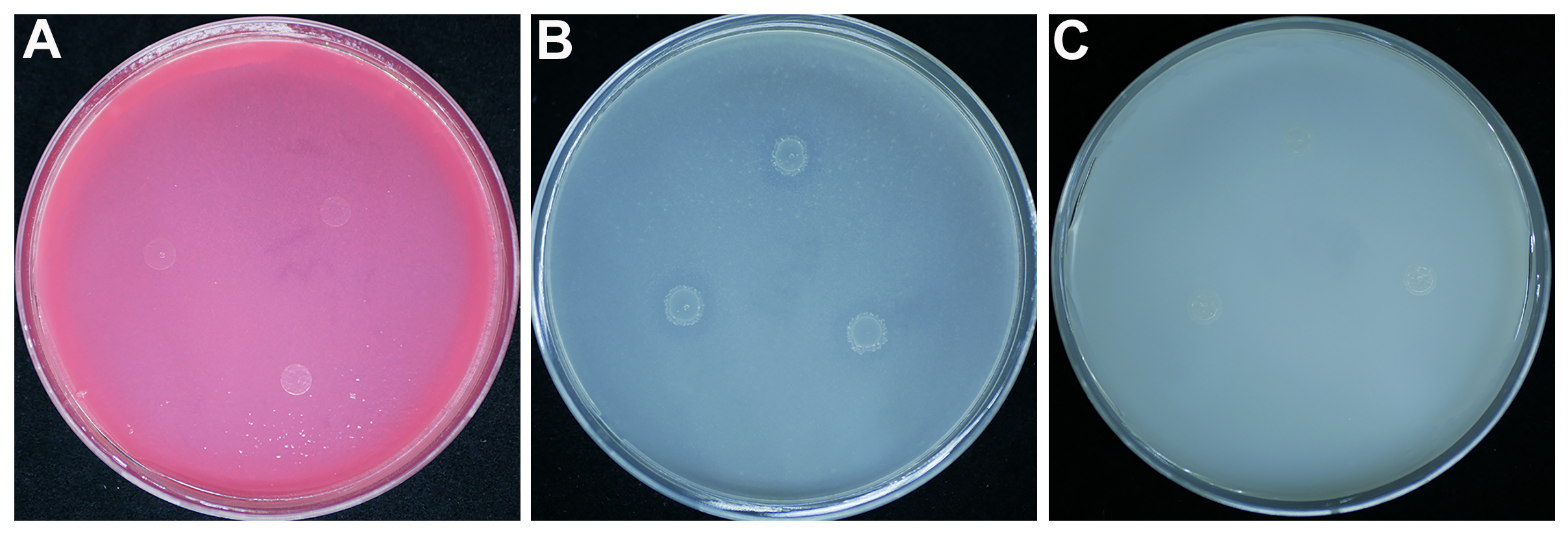

Supplement: Supplementary file 1 [file microorganisms-13-02701-s001.zip › Figure S1. Experimental results of AN6 on the degradation of cellulose, organic phosphorus-solubilizing and inorganic phosphorus-solubilizing..tif]
